# Improved resistance to ischemia and reperfusion, but impaired protection by ischemic preconditioning in patients with type 1 diabetes mellitus: a pilot study

**DOI:** 10.1186/1475-2840-11-124

**Published:** 2012-10-10

**Authors:** Richard Engbersen, Niels P Riksen, Marc J Mol, Bert Bravenboer, Otto C Boerman, Patrick Meijer, Wim JG Oyen, Cees Tack, Gerard A Rongen, Paul Smits

**Affiliations:** 1 Department of Pharmacology-Toxicology, Radboud University Nijmegen Medical Centre, P.O. Box 9101, Nijmegen, 6500 HB, The Netherlands; 2 General Internal Medicine, Nijmegen, The Netherlands; 3 Nuclear Medicine, Nijmegen, The Netherlands; 4 Department of Anesthesiology, Radboud University Nijmegen Medical Centre, Nijmegen, The Netherlands; 5 Slingeland Hospital, Doetinchem, Netherlands; 6 Canisius Wilhelmina Hospital, Nijmegen, The Netherlands; 7 Catharina Hospital, Eindhoven, the Netherlands

**Keywords:** Type 1 diabetes, Ischemia-reperfusion injury, Ischemic preconditioning, Hyperglycemia, Annexin A5

## Abstract

**Background:**

In patients with type 1 diabetes mellitus (T1DM), cardiovascular events are more common, and the outcome following a myocardial infarction is worse than in nondiabetic subjects. Ischemic or pharmacological preconditioning are powerful interventions to reduce ischemia reperfusion (IR)-injury. However, animal studies have shown that the presence of T1DM can limit these protective effects. Therefore, we aimed to study the protective effect of ischemic preconditioning in patients with T1DM, and to explore the role of plasma insulin and glucose on this effect.

**Methods:**

^99m^Technetium-annexin A5 scintigraphy was used to detect IR-injury. IR-injury was induced by unilateral forearm ischemic exercise. At reperfusion, Tc-annexin A5 was administered, and IR-injury was expressed as the percentage difference in radioactivity in the thenar muscle between the experimental and control arm 4 hours after reperfusion. 15 patients with T1DM were compared to 21 nondiabetic controls. The patients were studied twice, with or without ischemic preconditioning (10 minutes of forearm ischemia and reperfusion). Patients were studied in either normoglycemic hyperinsulinemic conditions (n = 8) or during hyperglycemic normoinsulinemia (n = 7). The controls were studied once either with (n = 8) or without (n = 13) ischemic preconditioning.

**Results:**

Patients with diabetes were less vulnerable to IR-injury than nondiabetic healthy controls (12.8 ± 2.4 and 11.0 ± 5.1% versus 27.5 ± 4.5% in controls; p < 0.05). The efficacy of ischemic preconditioning to reduce IR-injury, however, was lower in the patients and was even completely abolished during hyperglycemia.

**Conclusions:**

Patients with T1DM are more tolerant to forearm IR than healthy controls in our experimental model. The efficacy of ischemic preconditioning to limit IR-injury, however, is reduced by acute hyperglycemia.

**Trial Registration:**

The study is registered at www.clinicaltrials.gov (NCT00184821)

## Background

Type 1 diabetes mellitus (T1DM) substantially increases the risk for ischemic heart disease, including acute myocardial infarction [1]. In addition, the mortality rate in various clinical settings of ischemia-reperfusion (IR) injury, including acute myocardial infarction [2,3], and coronary artery bypass grafting [4,5], is higher in patients with diabetes mellitus, both in type 1 and in type 2 diabetic patients. Also, the incidence of heart failure is increased in patients with T1DM, and is directly associated with the glycated hemoglobin A_1c_ (HbA_1c_) concentration [6]. Therefore, novel therapies to reduce IR-injury and improve prognosis in patients with T1DM are urgently needed.

The most powerful intervention to limit myocardial infarct size, other than early coronary reperfusion, is ischemic preconditioning, which is defined as a reduction in infarct size by a preceding short period of myocardial ischemia [7]. Based on this principle, alternative cardioprotective strategies have been developed, including remote preconditioning and pharmacological preconditioning [8]. These interventions not only protect the heart against IR injury, but also other tissues including the kidney, the brain, the liver, and skeletal muscle. Unfortunately, it has been shown in animal models of myocardial infarction that various comorbidities, including hypercholesterolemia and diabetes, can limit the efficacy of these cardioprotective interventions [9]. In animal models of T1DM, studies on the effect of ischemic preconditioning on myocardial infarct size have yielded contradicting results [10].

In humans, the impact of T1DM on IR-injury and ischemic preconditioning has never been studied in vivo. Therefore, in the current study, we aimed to answer three research questions: 1) does T1DM affect the tolerance for IR?; 2) does T1DM modulate the protective effect of ischemic preconditioning?; and 3) is the effect of T1DM on these parameters dependent on the plasma glucose and insulin concentrations?

To this end, ^99m^technetium annexin A5 scintigraphy was used as a well-validated model of forearm IR-injury [11-15]. This model is based on the fact that early after reperfusion phosphotidylserin residues are exposed on the outer membrane leaflet of affected cells, as an early marker for cellular damage [16]. Annexin A5 binds with a high affinity to these residues. By labeling recombinant human annexin A5 to ^99m^Tc, these changes can be detected in humans in vivo as a marker for IR-injury.

## Materials and methods

### Ethics statement

The protocol has been approved by the Institutional Review Board of the Radboud University Nijmegen Medical Centre, and the study was performed in compliance with the recommendations of the Declaration of Helsinki. All patients signed for informed consent before participation. The study is registered at http://www.clinicaltrials.gov (NCT00184821).

### Subjects and experimental conditions

Fifteen male patients (aged > 18 years) with uncomplicated T1DM (defined as the absence of pancreatic beta cell reserve as reflected by a low plasma C-peptide) participated. Patients with hypertension (supine blood pressure >140/90 mmHg), diabetic retinopathy, (micro) albuminuria or cardiovascular disease were excluded. All subjects were studied after an overnight fast and 24 hours of caffeine abstinence.

The patients were randomized to either a normal glucose/high insulin group (n = 8) and high glucose/normal insulin group (n = 7). All patients lowered their evening dose of insulin dose by 30% at the night before the experimental days and skipped the morning dose of insulin to compensate for their fasting state. In eight patients, on the days of the experiments, blood glucose was tightly controlled between 4 and 8 mmol/l until the start of ischemia, using intravenous insulin when needed. In the remaining 7 patients, blood glucose was kept above 15 mmol/l at the start of ischemia, using intravenous glucose when needed. After one hour of reperfusion, all patients were allowed to have lunch and to use their normal insulin regimen. The results from these patients were compared with a control group of 21 healthy male subjects, who have been described in a previous study [11].

### Experimental protocol

All patients with T1DM were studied twice in a crossover design, with an interval of at least one week in between the experiments. In random order, each patient was subjected to the ischemic exercise protocol alone or to ischemic preconditioning followed by ischemic exercise. The healthy volunteers were subjected to either ischemic exercise alone (n = 13) or to ischemic preconditioning followed by ischemic exercise (n = 8).

In the ischemic exercise protocol, after cannulation of an antecubital vein of the dominant forearm, maximal voluntary contraction was determined in the non-dominant arm with a handgrip dynamometer (Baseline Hydrolic Hand Dynamometer, Fabrication Enterprise Inc., Irvington, New York, USA). Subsequently, the circulation of the non-dominant arm was occluded for 10 minutes by inflation of an upper arm cuff to 200 mmHg. Simultaneously, the subjects performed rhythmic isometric hand gripping exercise at 50% of maximal voluntary contraction for 5 seconds every 10-second period until exhaustion. The total duration of ischemia was 10 minutes. Immediately upon reperfusion, 0.1 mg of hydrazinonicotinamide (HYNIC)-derivatized recombinant human annexin A5, radiolabeled with 450 MBq Tc-99 m, was administered intravenously (5 mSv). Both hands were imaged at 4 hours after injection with a gamma camera (Siemens Orbiter, Hoffman Estates, Illinois, USA, equipped with low- energy high resolution collimators) connected to a Hermes Gold image processing system (Nuclear Diagnostics, Stockholm, Sweden) as previously described [11].

In the ischemic preconditioning experiments, the 10 minutes of ischemic exercise were preceded by 10 minutes of forearm ischemia (without concomitant handgripping) and 10 minutes of reperfusion.

### Preparation of ^99m^Tc-HYNIC-annexin A5

Radiolabeled annexin A5 was freshly prepared before each experiment by adding ^99m^Tc-pertechnetate (1500 MBq) in the presence of stannous tricine to succinimidylhydrazinonicotinamide (HYNIC)-conjugated recombinant human annexin A5 (NAS 2020, 0.275 mg per vial; Theseus Imaging Corp). For the patients with diabetes who were studied during hyperglycemia, recombinant human annexin A5 (obtained from Theseus Imaging Corporation) was conjugated with HYNIC in our own laboratory. The radiolabeling procedure of this HYNIC-conjugated annexin A5 was identical to that of NAS 2020.

### Analytical procedures

Plasma caffeine concentrations were determined by use of reversed-phase HPLC with UV detection set at 273 nm according to Schreiber-Deturmeny and Bruguerolle [17]. Insulin was measured by direct radioimmunoassay (RIA) using an International Standard for human Insulin (NIBSC code 83/500). The intra assay coefficient of variation (CV) was 5.6%, 6.6% and 6.7% at insulin concentrations of 20.5, 41.5, and 56.8 mE/L and the inter assay CVs at these concentrations were 9.8%, 10.0% and 13.6%. C-peptide was measured with a Double Antibody kit obtained from DPC (DPC Nederland B.V., Breda, the Netherlands).

### Data analysis and statistics

All the digitized gamma camera images were analyzed offline by the same investigator who was unaware of the experimental conditions (WJGO) using Hermes software (Hermes Gold, Nuclear Diagnostics, Stockholm, Sweden). Fixed-size circular regions of interest (ROI) were drawn over the thenar muscle of both hands. Special care was taken to avoid the major arteries and veins in the ROI. To correct for background activity, the final result was expressed as the percentage difference between the Annexin A5 uptake in the ROIs of the experimental hand and control hand (‘targeting’).

All results are expressed as mean ± SEM unless indicated otherwise. Between-group differences were assessed using a one-way ANOVA of variance, followed by unpaired t-tests to identify significant contrasts.

In the diabetes patients, the effect of ischemic preconditioning was analysed using a paired t-test. The effect of glycemic control during the experiment on the efficacy of ischemic preconditioning was statistically analyzed using a repeated measures ANOVA with ischemic preconditioning as a within-subject factor and the experimental condition as a between subject factor. Possible confounding by age was explored by determining the effect of the inclusion of age as a covariate in this statistical analysis. A two-sided p value <0.05 was considered to indicate statistical significance (SPSS for Windows, release 12.0.1).

## Results

Baseline characteristics of the study groups are summarized in Table 1. The plasma caffeine concentration was less than 0.5 mg/l in all subjects, indicating good compliance with caffeine abstinence. The plasma C-peptide concentration was ≤ 0.05 nmol/l in all diabetic patients, confirming a type 1 diabetes.

**Table 1:** Baseline characteristics of the subjects

	**T1DM; normal glucose**	**T1DM; high glucose**	**Healthy volunteers without IP**	**Healthy volunteers with IP**
N	8	7	13	8
Age (years)	28 ± 8	35 ± 11*	22 ± 3^‡^	24 ± 4^‡^
Body mass (kg)	78 ± 10	76 ± 8	74 ± 9	82 ± 7
Height (cm)	182 ± 7	178 ± 4	180 ± 7	185 ± 4
Systolic blood pressure (mmHg)	129 ± 9	124 ± 6	127 ± 7	131 ± 7
Diastolic blood pressure (mmHg)	78 ± 5	73 ± 8	75 ± 10	66 ± 10
Heart Rate (bpm)	72 ± 8	65 ± 6	68 ± 10	68 ± 14
HbA_1c_ (%)	7.6 ± 0.9	8.2 ± 0.9	-	-
Plasma glucose at onset of ischemic exercise (mmol/l)				
- IP^‡^	6.3 ± 1.2*^‡^	18.2 ± 1.5*^‡^	4.5 ± 0.6^‡^	
+ IP^‡^	6.4 ± 0.8^‡^	19.4 ± 2.0^‡^	-	5.0 ± 1.1^‡^
Plasma insuline at onset of ischemic exercise (mU/l)				
- IP^‡^	43.9 ± 27.6*^‡^	10.6 ± 4.2^‡^	14 ± 8^‡^	-
+ IP	28.3 ± 26.3	9.9 ± 4.2	-	-
Maximal voluntary contraction (kg)	49 ± 7^‡†^	40 ± 6^‡^	42 ± 9^†^	62 ± 12*^‡^

Values are means ± SD. *: p < 0.05 versus healthy controls without IP. ^†^:p < 0.05 versus healthy controls with IP. ^‡^:p < 0.05 versus T1DM with high glucose.

### Metabolic control during the experiment

As intended, the two groups of patients with diabetes significantly differed with respect to plasma insulin and glucose concentrations (see Table 1). Remarkably, in the patients who did not receive intravenous insulin treatment prior to ischemic exercise, the plasma insulin concentration did not significantly differ from the levels observed in the healthy volunteers.

The difference in plasma glucose concentration between the normoglycemic and hyperglycemic non-preconditioned diabetes groups persisted throughout the reperfusion period: 6.8 ± 1.9 (SD; n = 8) versus 16.4 ± 2.3 mmol/l (n = 7) at 1 hour and 11.6 ± 3.5 (n = 8) versus 19.3 ± 1.6 mmol/l (n = 7) at four hours after reperfusion for the group with and without extra insulin, respectively (p < 0.005 at both time points). After ischemic preconditioning followed by ischemic exercise, similar results were obtained: 6.8 ± 1.8 (n = 8) versus 19.0 ± 4.1 mmol/l (n = 7) at one hour and 12.5 ± 1.7 (n = 8) versus 22.2 ± 4.1 mmol/l (n = 7) at four hours after reperfusion (p < 0.001 at both time points).

### Annexin A5 targeting after ischemic exercise

**Figure 1:**
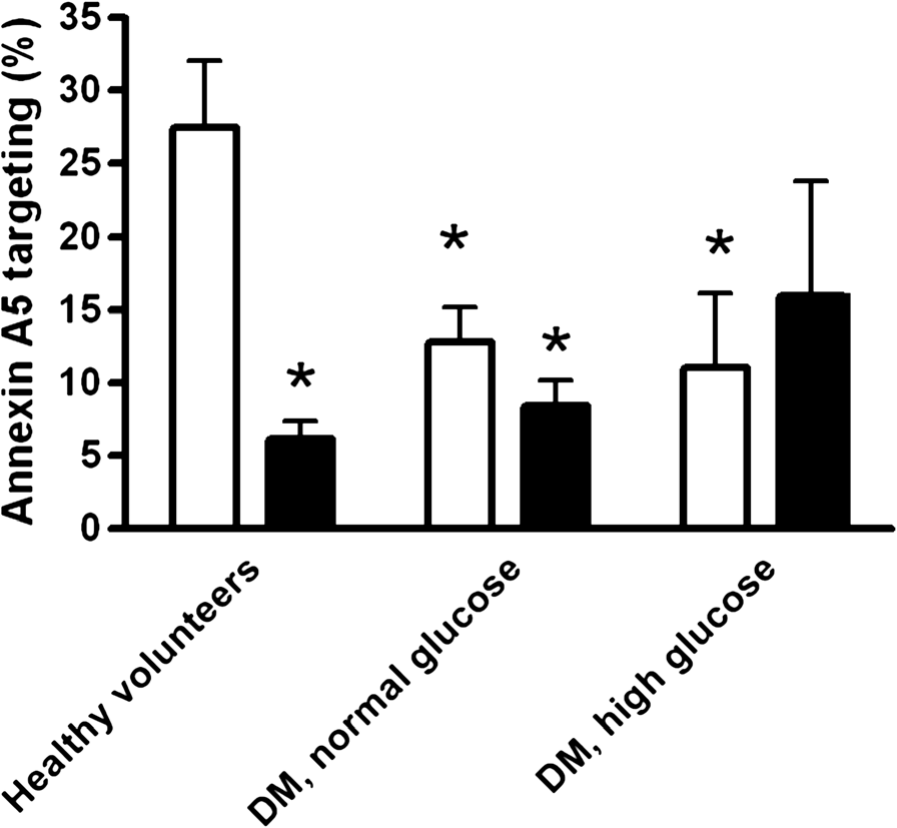
**Targeting of annexin A5 to the thenar muscle after ischemic exercise at four hours after reperfusion in the absence (open bars) and presence (black bars) of ischemic preconditioning.** *: p < 0.05 versus healthy volunteers without ischemic preconditioning.

**Figure 2:**
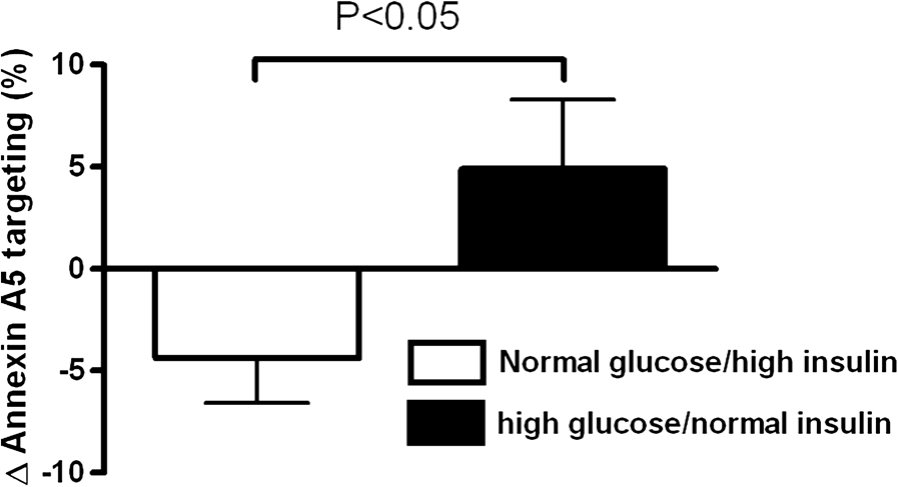
**Effect of ischemic preconditioning on annexin A5 targeting in the two experimental conditions in patients with diabetes (normal glucose and high glucose).** Δ targeting was calculated as the targeting after ischemic exercise with preconditioning minus targeting after ischemic exercise without preconditioning.

Independent of the metabolic control during the experiment, annexin A5 targeting after ischemic exercise was lower in patients with T1DM than in healthy volunteers (12.8 ± 2.4 (SEM; n = 8) and 11.0 ± 5.1% (n = 7) in the two groups of patients with diabetes versus 27.5 ± 4.5% (n = 13) in the healthy controls; p < 0.05 for diabetes versus control; Figure 1). Ischemic preconditioning tended to reduce targeting in the patients with normal glucose/high insulin without reaching statistical significance (12.8 ± 2.4 versus 8.4 ± 1.8%; p = 0.087; n = 8). In the patients who were studied under hyperglycemic conditions, ischemic preconditioning did not limit targeting (11.0 ± 5.1 versus 15.9 ± 7.9% in absence and presence of ischemic preconditioning, respectively; p = 0.20; n = 7). Within the tested population of patients with diabetes, the protective effect of ischemic preconditioning was significantly higher in those who were studied under normoglycemic hyperinsulinemic conditions as compared with those who were studied during hyperglycemia (repeated measures ANOVA: significant interaction between the effect of ischemic preconditioning and experimental condition (p = 0.035 and p = 0.025 without and with age as a covariate respectively); Figure 2). In contrast to the patients with diabetes, the healthy volunteers showed a profound and consistent reduction in annexin targeting by ischemic preconditioning (Figure 1).

## Discussion

The current study is the first to investigate in humans in vivo whether T1DM affects the tolerance for IR and the protection by ischemic preconditioning. Patients with T1DM appear to be more resistant to IR in the forearm skeletal muscle. The efficacy of ischemic preconditioniong to limit IR-injury, however, is lower in patients with diabetes than in nondiabetic control subjects, and is even completely abolished during hyperglycemia.

Despite optimal reperfusion therapy, morbidity and mortality in patients suffering an acute myocardial infarction remain significant [18]. Therefore, novel strategies to limit IR-injury are warranted. Based on the finding that ischemic preconditioning profoundly reduces myocardial infarct size, several novel treatment modalities have recently been developed and tested in humans, including remote preconditioniong [19], and pharmacological preconditioning [8]. For patients with diabetes, this need for additional treatment is even more urgent, given the fact that the risk of cardiovascular events is higher, and the mortality rate following a cardiovascular event is increased, both in type 1 and in type 2 diabetic patients [2,3]. In addition, the incidence of heart failure is higher in patients with T1DM [6]. There are several potential explanations for this worse outcome following infarction in patients with diabetes. In diabetic patients suffering from a myocardial infarction, infarct size (estimated with nuclear imaging) is larger than in nondiabetic patients, both after thrombolysis and after primary percutaneous coronary intervention [20,21]. It was suggested, however, that the small difference in infarct size could not completely account for the 4–6 fold increased mortality in the diabetic patients in this study [21]. This was recently confirmed in an animal study, in which the mortality rate following a myocardial infarction was higher in diabetic rats than in control rats, despite similar infarct size. It was speculated that autonomic dysfunction in the diabetic rats contributes to this increased mortality [22].

Despite the clinical observations that suggest that patients with diabetes are more susceptible to myocardial IR-injury, animal studies have provided conflicting results. A vast amount of studies have explored the tolerance of myocardial tissue for IR in animal models of type 1 and type 2 diabetes mellitus [9,10,23-27]. Although some studies have reported that the myocardium is more resistant to IR-injury in animals with T1DM, other studies have found either no effect, or an increased susceptibility. Probably, this tolerance to IR is dependent on the duration of diabetes (an increased resistance has been reported in particular early after the onset of diabetes), the animal species, and the duration of IR [10]. In addition, it has to be taken into account that the animal models of T1DM do not accurately reflect the human pathology of diabetes in all aspects (e.g. the toxins administered to destruct the pancreas might have alternative mechanisms of action, and do not reflect the auto-immune destruction that occurs in humans).

Experiments in animal models of myocardial infarction have demonstrated that comorbidities, including diabetes, myocardial hypertrophy, and hypercholesterolemia can limit the protective effect of (ischemic) preconditioning [9]. Several animal studies have investigated whether the diabetic heart is still amenable to the cardioprotective effect of (ischemic) preconditioning. Most studies in animal models of T1DM (mainly diabetes induced by the administration of streptozotocin) have demonstrated that the cardioprotective effect of ischemic preconditioning is abolished [28,29]. Also, acute hyperglycemia completely abolished the infarct size-limiting effect of ischemic precondioning [30]. In Goto-Kakizaki diabetic rats, the cardioprotective effect of preconditioning could be restored, however, by increasing the intensity of the preconditioning stimulus, illustrating that diabetes increases the threshold for preconditioning [31]. It has been suggested that the diminished potential for cardioprotection in diabetes is due to impaired function of the ATP-dependent potassium channel (K_ATP_-channel), or due to decreased phosphorylation of important signalling kinases including Akt and glycogen synthase kinase (GSK)-3β [10,26].

These findings of a reduced efficacy of ischemic preconditioning have been confirmed in experimental studies on IR-injury in human atrial trabeculae: in patients with diabetes, the cardioprotective effect of ischemic preconditioning was either abolished, or the threshold for cardioprotection was increased, possibly due to impaired opening of K_ATP_-channels [32-34].

In our study, annexin A5 targeting after forearm IR was lower in patients with T1DM than in nondiabetic control subjects, indicating an increased resistance to IR. We postulate that this is, at least in part, due to the protective effect of insulin against IR-injury. The patients with diabetes and strict administration of insulin during the experiment only marginally differed from healthy controls with respect to the plasma glucose concentration while their plasma insulin concentration was significantly higher. These two groups did not differ at baseline in other respects. Therefore, we propose that the higher circulating insulin contributes to the observed tolerance to forearm IR in this group. This conclusion is supported by a large body of preclinical evidence indicating a protective effect of insulin against IR-induced cell death [35-37]. Based on this preclinical evidence, we expected an increased targeting of annexin A5 after ischemic exercise in the patients with T1DM who were studied during hyperglycemic conditions. This, however, was not observed. We postulate that this is because the protective effect of chronic insulin treatment was still present after skipping one dose of insulin and remained effective during the experiments.

Despite this reduced susceptibility for IR-injury, additional protection by ischemic preconditioning was much less effective in patients with T1DM than in healthy control subjects. In more detail, this protective effect was small, and only observed in patients during normoglycemic conditions. During hyperglycemia, ischemic preconditioning did not reduce annexin A5 targeting at all. In the patients who were studied during hyperglycemia, the plasma insulin concentration did not differ from that observed in healthy volunteers. Therefore, it is unlikely that insulin affected the protective effect of preconditioning in these patients, and it is more likely that the hyperglycemia abolished this protective effect. This is supported by previous preclinical observations in animals using myocardial infarct size as an endpoint [30,38] and it is consistent with epidemiological evidence in humans [39]. A possible explanation for this phenomenon is an acute impairment of mitochondrial K_ATP_-channels in response to hyperglycemia [38]. The patients who were studied during hyperglycemia were significantly older than the healthy volunteers and the patients who were studied during normoglycemia. Advanced age has been associated with a reduced efficacy of ischemic preconditioning to protect against ischemic cell death [9]. However, the interaction between ischemic preconditioning and experimental condition remained when age was incorporated as a covariate in the analysis of variance, excluding that age is a significant confounder.

Taken together, these data provide human experimental evidence for aggressive normalization of plasma glucose in patients with T1DM who experience repeated bouts of ischemia, such as angina pectoris or transient ischemic attacks, in order to optimize benefit from endogenous protection by ischemic preconditioning. These observations fit well with clinical data suggesting benefit from insulin treatment in critically ill patients with a disturbed tissue perfusion [40].

There are several limitations of our study. Since targeting of annexin A5 after forearm IR was low in patients with diabetes, the potential window of protection by ischemic preconditioning was smaller. This could have affected the power of our study to detect an effect of ischemic preconditioning in these patients. Indeed, in the patients who were studied in normoglycemic conditions, ischemic preconditioning tended to reduce targeting: in 6 out of 8 volunteers targeting was reduced (p = 0.087). Our failure to detect a significant protection by ischemic preconditioning in this group of patients might therefore result from a lack of power due to reduced targeting at baseline. Despite this small window of opportunity to further reduce annexin A5 targeting, in the patients with type 1 diabetes the effect of ischemic preconditioning was significantly affected by the experimental condition (hyperglycemic/normoinsulinemic or normoglycemic/hyperinsulinemic), consistent with a direct effect of hyperglycemia on the efficacy of ischemic preconditioning to prevent targeting of annexin A5. Secondly, since we did not use a hyperinsulinemic clamp technique, serum glucose levels tended to increase in the normoglycemic group during the last hours of the reperfusion period. Furthermore, our experimental design resulted in differences in both serum glucose and insulin between the two groups of patients with diabetes, complicating the interpretation of the study. However, the current design was chosen to allow an optimal comparison between the healthy volunteers and the normoinsulinemic hyperglycemic patients with diabetes.

## Conclusions

In conclusion, our data indicate that patients with T1DM who are well controlled with chronic insulin treatment appear to be less vulnerable to IR-injury in our experimental model compared to healthy control subjects. Most probably, this is caused by the higher plasma insulin concentration, which induces protection. In addition, hyperglycemia abolishes the protective effect of ischemic preconditioning on IR-injury. Taken together, these findings provide experimental evidence in humans to support aggressive normalization of plasma glucose using insulin in patients with T1DM who experience repeated bouts of ischemia, in order to optimize benefit from insulin-induced protection and endogenous protection by ischemic preconditioning.

## Competing interests

The authors declare that they have no competing interests.

## Authors’ contributions

RE participated in the design of the study, carried out the study, performed the analyses, and drafted the manuscript. NPR participated in the analyses of the results, and drafted the manuscript. MJM and BB helped in the patient recruitment and critically reviewed the manuscript. PM participated in performing the experiments. OCB and WJGO performed the analyses of the nuclear studies and critically reviewed the manuscript. PS, GAR, and CT participated in the design of the study and critical review of the manuscript. All authors read and approved the final manuscript.
